# The effect of ISO/IEC 27001 standard over open-source intelligence

**DOI:** 10.7717/peerj-cs.810

**Published:** 2022-01-06

**Authors:** Abdallah Qusef, Hamzeh Alkilani

**Affiliations:** 1 Software Engineering Department, Princess Sumaya University for Technology, Amman, Jordan; 2 Computer Science Department, Princess Sumaya University for Technology, Amman, Jordan

**Keywords:** Cybercrimes, Open data, Open-source intelligence, OSNIT, Reconnaissance

## Abstract

The Internet’s emergence as a global communication medium has dramatically expanded the volume of content that is freely accessible. Through using this information, open-source intelligence (OSINT) seeks to meet basic intelligence requirements. Although open-source information has historically been synonymous with strategic intelligence, today’s consumers range from governments to corporations to everyday people. This paper aimed to describe open-source intelligence and to show how to use a few OSINT resources. In this article, OSINT (a combination of public information, social engineering, open-source information, and internet information) was examined to define the present situation further, and suggestions were made as to what could happen in the future. OSINT is gaining prominence, and its application is spreading into different areas. The primary difficulty with OSINT is separating relevant bits from large volumes of details. Thus, this paper proposed and illustrated three OSINT alternatives, demonstrating their existence and distinguishing characteristics. The solution analysis took the form of a presentation evaluation, during which the usage and effects of selected OSINT solutions were reported and observed. The paper’s results demonstrate the breadth and dispersion of OSINT solutions. The mechanism by which OSINT data searches are returned varies greatly between solutions. Combining data from numerous OSINT solutions to produce a detailed summary and interpretation involves work and the use of multiple disjointed solutions, both of which are manual. Visualization of results is anticipated to be a potential theme in the production of OSINT solutions. Individuals’ data search and analysis abilities are another trend worth following, whether to optimize the productivity of currently accessible OSINT solutions or to create more advanced OSINT solutions in the future.

## Introduction

The digital forensic investigation may uncover substantial and incriminating evidence. Still, if they cannot communicate it to a lay audience in a clear and intelligible manner, the case may be lost (Montasari, 2017). Investigators must describe the process to a court and jury because of the intricacy of the tools and techniques utilized in conducting a digital investigation. These instruments and techniques must also meet specific standards of practice and be approved by other field investigators. Despite this, there is still a lack of agreement and formal process models in the field of digital forensics that courts may use to assess the trustworthiness of digital evidence submitted before them (Montasari, 2018).

The word “open source” applies explicitly to content that is freely accessible to the public. If access to a piece of knowledge requires specialized expertise, software, or methods, it cannot correctly be called open source. Notably, open-source content is not restricted to what can be found through popular search engines. Though web pages and other Google-accessible sites are essential sources of open-source content, they are far from the only ones.

- A new mass communication mode recognized as networking sites has arisen (Al Mutawa, Baggili & Marrington, 2012). Former Google CEO Eric Schmidt estimated that the Internet would split into Chinese-led and US-led models over the next decade.
- In Sundar Pichai’s leadership, Google investigated the possibility of launching a restricted model of its search engine in China, sparking internal and external debate.

In January 2021, there will be 4.66 billion people globally, accounting for 59.5% of the global population. 92.6% (4.32 billion) of this total access to the network through mobile devices.

China, India, and the United States led all other nations in terms of online users. China has over 854 million web users, while India has about 560 million. In reality, both countries have sizable populations that are disaffected.

After the Cold War, human society was made more open, and the turmoil and universal usage of the Internet made the world smaller. The bulk of the populace now uses the Internet to mix, read, chat to mates, share data and information, phone, and divert attention. Statista estimates that in April 2020, global internet users accounted for 4.57 billion individuals or 59% of the global population. According to online protection adventures, Internet users are expected to reach 7.5 billion by 2030, accounting for 90% of the total population. Many users will practice what is currently known as the Internet due to the Internet’s risky expansion and shares development.

As worldwide social orders move consistently towards digitalization, a considerable measure of computerized information will be created because of the communications of individuals and organizations on the Internet. A decent level of this information is freely accessible. Realizing how to utilize it opens up numerous chances for associations to grow their exercises and work productively in the present data age.

Furthermore, there is an assortment of freely usable information accessible online assets instead of ordinary web indexes. We will go into this in more depth later; however, Shodan and Censys might be utilized to discover IP addresses, workers, open ports, webcams, and printers, among numerous other web things.

Open-source intelligence (OSINT) is a concept that refers to any publicly available data used to fulfill a specific need for information. OSINT properties may be isolated or web-based in two systems. Still, since the environment is growing digitalized, most OSINT experience is taken from Internet properties.

Open-source intelligence (OSINT) collects and reviews freely accessible data, mainly from web media. The vast volume of digital data is generally recognized as the most complicated feature of any OSINT collection operation. Interestingly, there are various OSINT resources and strategies available to assist OSINT assemblies with this mission. On the other side, governments and law enforcement authorities use it to combat violence and extremism, forecast global developments and deliver timely and actionable intelligence to lawmakers to allow them to make educated decisions.

**Figure 1:**
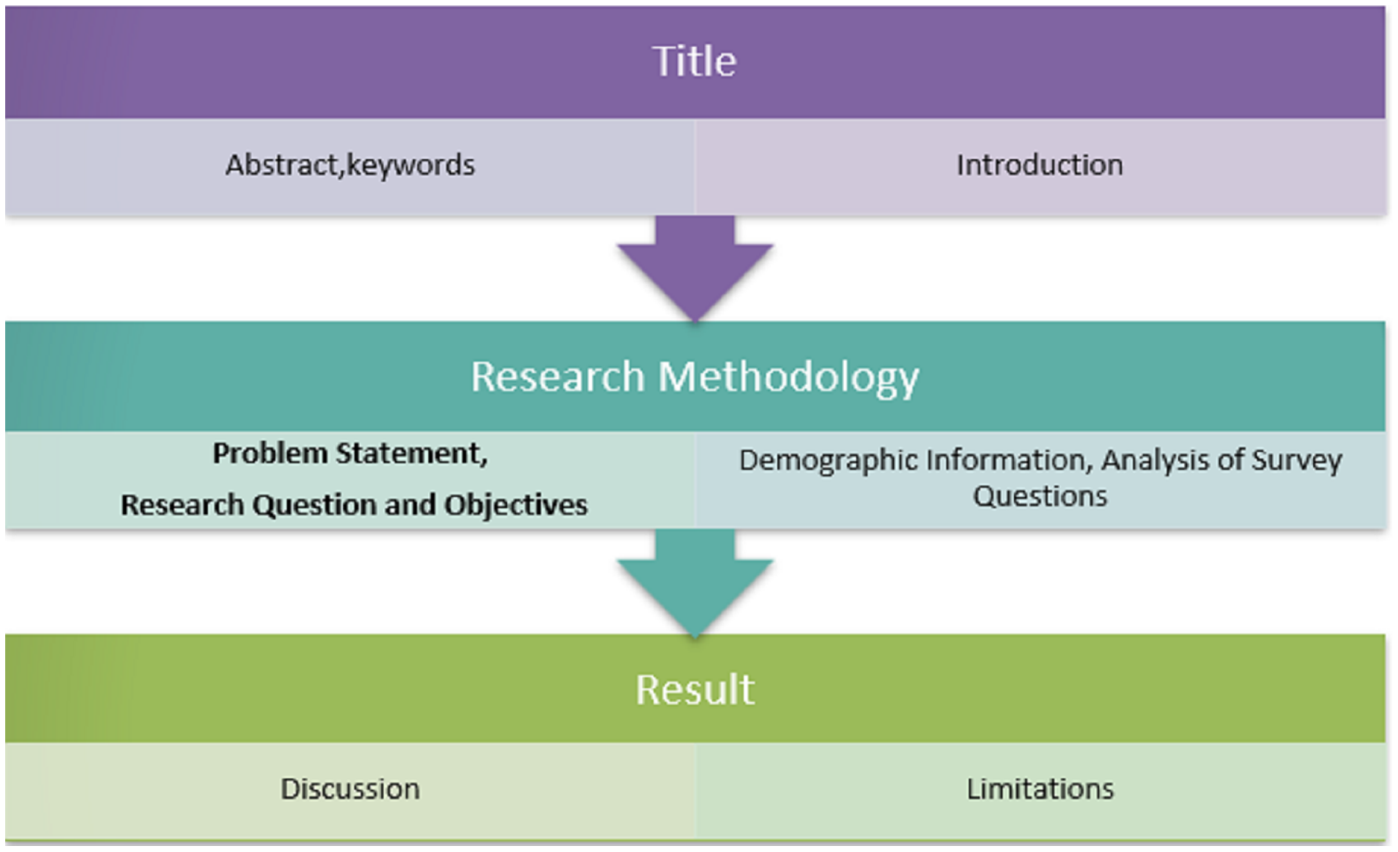
Structure of the paper.

OSINT techniques are used throughout the cybersecurity domain to uncover stolen data and to locate flaws in information management networks, in addition to their vital position in counterintelligence operations (identify and counter threats to IT systems originating from cyberspace), shown the structure of the paper in Fig. 1.

OSINT, according to Steele (2006), is unclassified knowledge that is purposefully found, thereby categorizes, separates, and communicates to a small audience to answer a particular query. Concerning the utilization of OSINT for removing social assessment and feelings, Santarcangelo et al. (2015) proposed a model for deciding client sentiments about a given catchphrase through interpersonal organizations, explicitly contemplating the descriptive words, intensifiers, and invalidations utilized in tweets. Shockingly, it is an essential catchphrase-based arrangement just intended for the Italian language, not considering semantic issues. On the other hand, Kandias et al. (2017) could relate individuals’ utilization of informal organizations (specifically, Facebook) to their pressure level. In any case, the examinations were completed distinctly with 405 clients, while these days, there is an opportunity of handling a lot of more significant measures of information. Another intriguing investigation is led in Senekal & Kotzé (2019), where creators applied Natural Language Handling (NLP) to WhatsApp messages to potentially forestall the event of mass savagery in South Africa. Shockingly, the examination is restricted to instant messages, consequently barring essential data revealed through media material.

A few works investigate the use of OSINT for criminal examinations corresponding to cybercrime and coordinated wrongdoing (Kao et al., 2018). For instance, OSINT could increment the precision of arraignments and captures of guilty parties with systems like the one proposed by Quick & Choo (2018). Solidly, creators apply OSINT to advanced legal information of various gadgets to improve the crook insight examination. In this field, another chance that OSINT yields are the identification of illicit activities just as the anticipation of future wrongdoings, for example, militant psychological assaults, murders, or, then again, assaults.

The European tasks ePOOLICE (Pastor & Larsen, 2017) and Escapade (Aliprandi et al., 2014) were intended to create compelling models for checking open information examine the society identify arising coordinated wrongdoing. Rather than the past, referenced ventures, whose propositions were most certainly not utilized in genuine cases, Delavallade, Bertrand & Thouvenot (2017), depict a model dependent on informal communities’ information that can remove future wrongdoing pointers. Such a model is then applied to the copper robbery and the jihadist purposeful publicity use cases.

Right now, there is a broadening of administrations offered on the web, which has prompted the development of a developing mass of computerized information (Pune, 2020). This information can be gotten to by Application Programming Interfaces (APIs), or various administrations, applications, and so forth. For instance, individuals who utilize administrations accessible on the web to enlist destinations of interest, travel, political or strict affiliations, photographs, among different wellsprings of exposure of a rational public nature, can be found. In any case, not every person knows that a massive extent of this data is freely uncovered and can be utilized by people or associations with various purposes (Pastorino, 2019). This implies that all data distributed on informal organizations, conversation discussions, and gathering visits, among different sources, is free and available to anybody, considering the limitations that may apply (Passi, 2018). In any case, in any event, when a lot of information is discovered, these are themselves considered unevaluated material acquired from any source. However, when such information is expounded and treated, it becomes data by procuring significance and utility.

Moreover, if experience, comprehension, and codification are added to this, such data becomes information. Whenever this is made accessible to an individual inspired by the motivation behind aiding the dynamic interaction, knowledge happens (Portillo & Nykiel, 2019; Pastor-Galindo et al., 2020; Norton, 2011). The exercises of get-together and relating such data using apparatuses are called Open-source insight (OSINT) (Norton, 2011). Although this work of gathering and corresponding data is certainly not a new action, some more contemporary meanings of OSINT can be given. “Unclassified data that has been purposely found, segregated, refined and spread to a select crowd to address a particular inquiry” (NATO, 2001; Korkisch, 2010). “Insight that is delivered from openly accessible data and is gotten utilized and scattered in a convenient way to a suitable crowd to react to a particular knowledge demand” US DoD (Department of Defense) (LISA Institute, 2020). “Gather, interaction and relate public data from open information sources, for example, the media, interpersonal organizations, discussions, and sites, public government information, distributions or business information” (Pastor-Galindo et al., 2020).

As per Carcaño (2018), OSINT sources are public sources—autonomous of whether their substance is marketed or accessible. They can be archives with any substance, in any medium, under any methods for transmission or method of access. OSINT sources are recognized from different types of knowledge since they should be lawfully open to the general population without disregarding copyright or security laws (Hassan, 2018). These open-sources incorporate (Khera, 2020; Richelson, 2016; Williams & Blum, 2018): • Government information and public reports, spending plans, hearings, phone registries, question and answer sessions, sites, and talks; • Expert and scholastic distributions, data gained from diaries, gatherings, symposia, academic papers, expositions, and theories; • Business information and pictures, monetary and modern assessments, just as data sets; • Grey literature, specialized reports, preprints, licenses, working archives, business records, unpublished works, and notices; • Photographs and recordings, including metadata; • Geospatial data (*e.g*., guides and business imaging items). Then again, open-source data is not restricted to the principle web indexes (Recorded Future, 2019). Indeed, playing out a pursuit on any motor produces gigantic data sources far from the solitary sources given. On the web, there are various open-hotspot for various sorts of searches: recordings, pictures, messages, and so forth.

As per AlKilani & Qusef (2021), the authors proposed an integration process between selected OSINT techniques and ISO 27001 standard in some relevant clauses for additional security. They presented a set of OSINT techniques that deals with background checks and screening new hires in addition to vendor’s risk assessment in the context of integration them with the international standard ISO 27001.

### Sources for OSINT

Open-source information (OSINT) has existed since the introduction of mass technology, such as publications and official announcements for intelligence collection, primarily by the military. However, it is now still often used by other organizations. The demand for OSINT overgrew as the Internet spread and made more uncontrolled intelligence sources available (Glassman & Kang, 2012). OSINT may be delivered by printed or interactive channels, such as magazines, television, journals, and, more recently, internet material. Security analysts have used OSINT to complement sensitive information (Best & Cumming, 2007).

**Figure 2:**
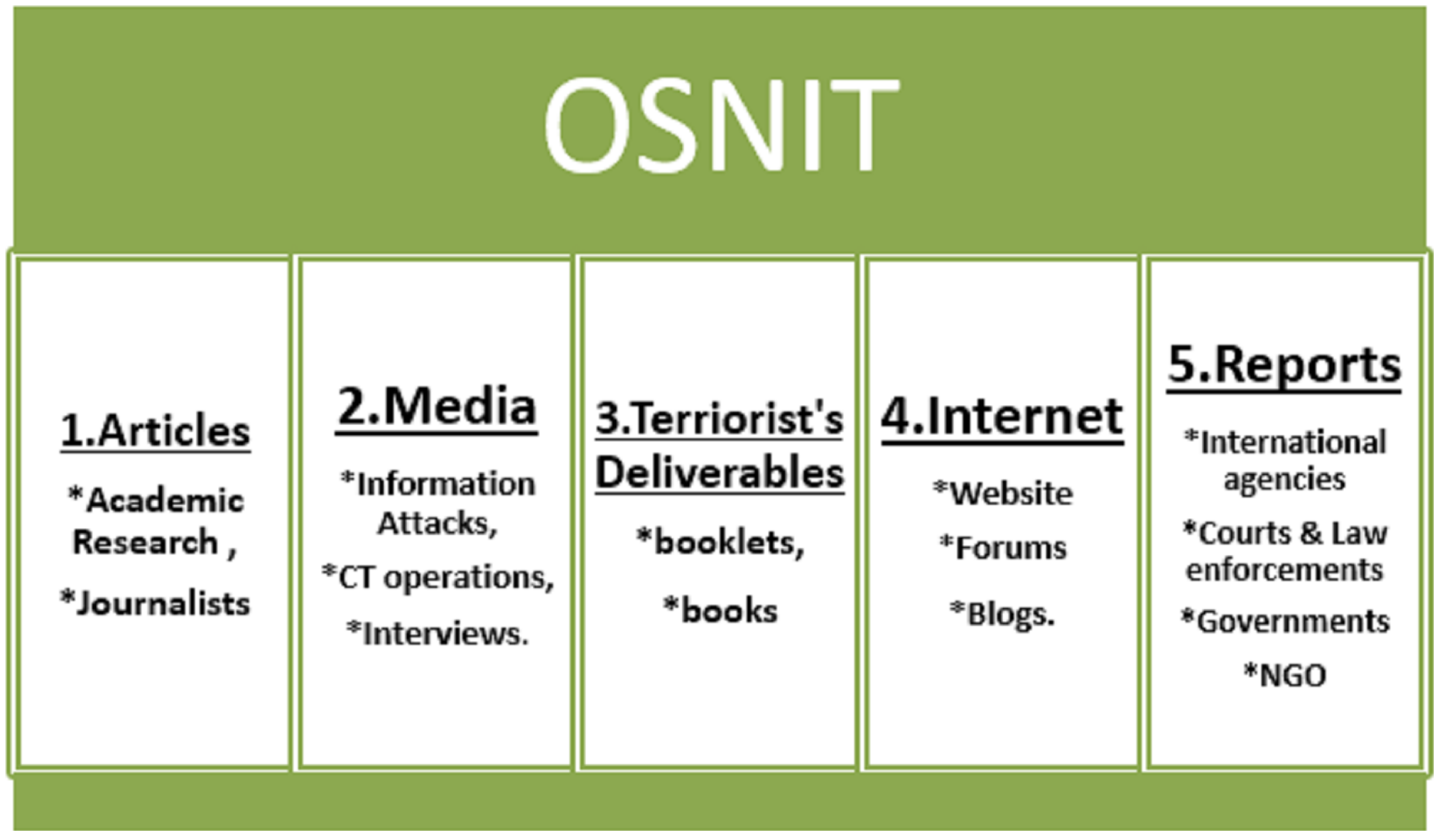
OSNIT various sources.

The Internet has been a valuable platform for every intelligence analyst owing to Internet filtering, scanning, indexing, and search engines (Appel, 2011). The overwhelming number of citizens have Internet and digital platforms, meaning that many archives of personal minds are preserved in the Internet’s official, semi-public and large websites (Appel, 2011). According to Koops, Hoepman & Leenes (2013), law enforcement authorities mine Facebook and Twitter for intelligence purposes. Online news sites are often tracked to track and deter extremist violence see Fig. 2.

### Who needs OSINT?

Open-source intelligence is more often associated with military intelligence and organizations. The OSINT user base is far broader. OSINT is primarily used by large global corporations, banks, and diverse sectors to collect information and market analysis for decision making, strategic advantage, and business security.

- Government/Government bodies, including defence, intelligence, and law enforcement agencies
- International organizations
- Business companies
- Penetration testers, hackers
- Individuals who value their privacy.

#### Government agencies and intelligence services

OSINT has the vital capacity to strengthen law enforcement agencies’ know-how and other security bodies in fighting crimes and safeguard the population, authorities, networks, institutions, public administrators of criminal organizations, extremism, and other cyber-related assaults. Because the bulk of Internet users have at minimum one web-based media account, a vast number of individual data may be collected from the Internet to collect intelligence about suspects, take into account their views, and detect possible perpetrators when they perform crimes. In reality, an analysis of OSINT results also resolves the power evaluation.

#### Terrorist organizations and black hat hackers

Criminals and black hat hackers use OSINT plans and techniques similar to those utilized by law-abiding people to obtain intelligence on goals before committing a crime. Social engineering attacks begin with adequate data about the goal. The gathered data is utilized to redo the assault as indicated by every individual, expanding the opportunity for effective interruption—the different periods of the Miter ATT&CK digital murder chain.

**Figure 3:**
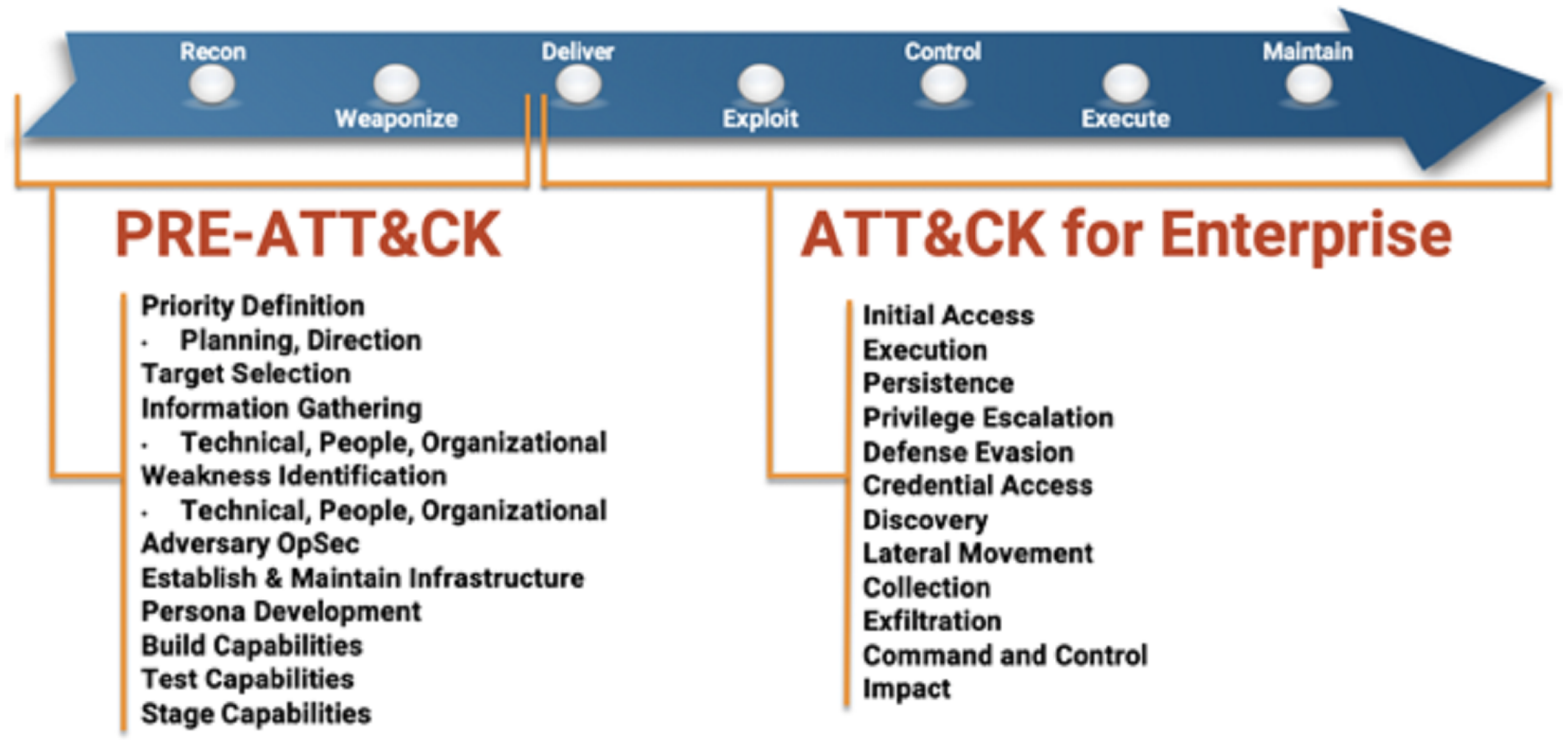
Various phases of the MITRE ATT&CK cyber kill chain.

As shown in Fig. 3, the MITRE ATT&CK kill chain, most cyberattacks begin with a reconnaissance process, including OSINT.

### OSINT as a business tool

OSINT is well developed as more private-sector technology and solutions are used. The efficacy and importance of OSINT are no more in doubt; instead, the concern is how effectively it can be integrated into a specialization for security experts in the public and private sectors (Gibson, 2004). Table 1 shows OSINT Source Checklist. Integrating OSINT systems into corporate structures can result in significant gains and improve the organization’s overall effectiveness in responding to internal and external challenges. We may appreciate the significant attributes.

**Table 1:** OSINT source checklist (taken from Gibson, 2004).

Checklist	Description
Authority	Are the OSINT origins held in high regard by peers or consumers?
Accuracy	For reference, how reliable is the OSINT source, and can it be checked or evaluated?
Objectivity	Seems to be the OSINT root in some manner distorted?
Timely	Is the OSINT root timestamped, date-stamped, or geolocated?
Relevancy	How essential is the OSINT element?

#### Case study 1: The Cambridge Analytica scandal

The Cambridge Analytica scandal has shocked the nation. Facebook has been sued in England and Wales for “losing control” of the details of over a million people.

The suspected shortcomings were exposed during the Cambridge Analytica scandal, in which collected data were used for election-related advertisements. Journalist Peter Jukes, who is spearheading the campaign, asserts that his database has been hacked. According to Facebook, there was “no proof” that data from UK or EU accounts had been passed to Cambridge Analytica. However, the internet giant’s lawsuit, likely to take at least 3 years, may contend that a “loss of ownership” over consumers’ sensitive data demands individual payments. The collection of sensitive details from Facebook users through third-party applications was at the heart of the Cambridge Analytica privacy controversy that broke in 2018. Cambridge Analytica’s software on Facebook extracted data from users that communicated with it - as well as non-consenting contacts. As per the petition, Facebook gathered data without authorization, which violates the Data Protection Act 1998. For its involvement in the Cambridge Analytica fiasco, Facebook was fined £500,000 by the UK’s cybersecurity agency in October 2018. According to the Information Commissioner’s Office (ICO), Facebook helped facilitate an “extreme violation” of the rule. Facebook issued an apology and allowed users to see the “blacklisted applications” that had obtained their information. There is precedence for such a large-scale court action in the United States, although none exists in the United Kingdom. Google decided to pay a reported $22.5 million (£16.8 million) in a 2012 lawsuit filed by the United States Federal Trade Commission (FTC). Also, a limited number of British clients reached an out-of-court agreement with the firm.

#### Case study 2: WhatsApp controversy highlights growing fears about data privacy

Many users left WhatsApp after the messenger service modified its privacy policies. Concerns that their data could end up with Facebook drive them to rivals seeking better data security. According to smartphone app monitoring company Sensor Tower, WhatsApp’s rival Signal saw 17.8 million installs between January 5 and January 12, up from just 285,000 last week. Simultaneously, fellow chat app Telegram saw 15.7 million installs, double the 7.6 million downloads it saw the week before. Many have migrated to the lesser-known Threema, a paying messaging app that mainly serves German-speaking countries where cybersecurity is strong.

### Significant benefits of open-source intelligence

For both types of organizations, the advantages of OSINT are self-evident. The subsequent segment addresses the most significant matters:

1. OSINT is strategically advantageous in contrast to other forms of traditional intelligence collection, such as Signals Intelligence (SIGINT) or Geospatial Intelligence (GEOINT), which have a poor cost of return on investment (ROI). Without spending a lot on consultants and other high-priced tools, businesses with small surveillance expenses may employ OSINT tactics or subsidize it to a third-party provider.
2. OSINT databases are abundant and provide material about almost every topic imaginable and accessed at any time. For instance, a regular Google search just covers the basics of Google’s indexed page material. OSINT uses the site’s three layers (surface, underground, and darknet) to ensure that the quest covers nearly all web material.
3. By doing an OSINT information leakage evaluation, OSINT tools may uncover flaws in IT applications. A company may detect compromised user passwords, accessible ports, vulnerable network networks, and already-in-use expired applications and operating system models.

### How Is open-source intelligence used?

**Figure 4:**
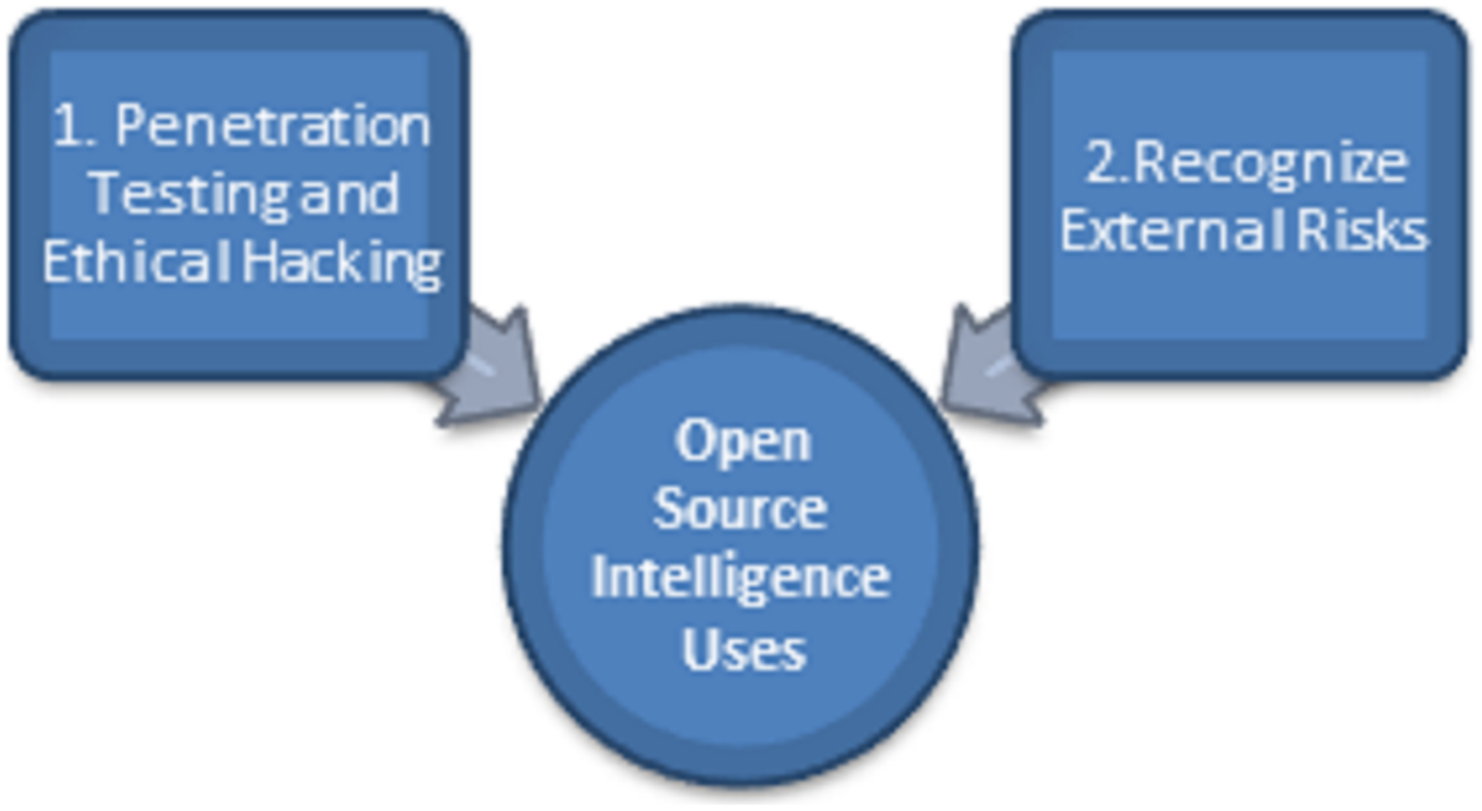
Primary uses of open-source Intelligence.

Since exploring the fundamentals of Open-source intelligence, we will explore how it is widely utilized in cybersecurity. We commonly use these 2 cases, as shown in Fig. 4:

#### Penetration testing and ethical hacking

Security experts use open-source information to detect possible vulnerabilities in social networks and remediate them before being targeted by threat actors. Frequently identified bugs include the following:

- Accidental disclosures of confidential details, for example, through social media,
- Ports that are left open or computers that are linked to the Internet that are not protected,
- Unpatched applications, such as websites operating obsolete versions of current content management systems,
- Resources that have been leaked or revealed, such as patented code on paste bins.

#### Recognize external risks

Internet is an excellent source of vulnerability awareness; enumerating the new weaknesses being abused empowers IT and protection practitioners to allocate their time and money to address current issues.

Typically, this form of work involves several data points to recognize and prove a hazard; for, *e.g*., while a single person tweeting does not warrant consideration, whether it is understood to be connected to a particular sector, the same entity transmitting several threats may deserve scrutiny.

It is crucial to note that Open-source information is commonly worked in tandem with other forms of intelligence. Most knowledge obtained by telemetry, dark network, and outside networks is used to validate and build public information. There are several means of doing these analyses, which we will take a look at later.

### What constitutes open-source information?

When knowledge or details in total is:

- Intended for a broad audience (*e.g*., news channels and newspaper contents).
- Open to the public at no charge.
- Open to the public upon paying a premium (*e.g*., Books, Journals, Magazines, digital libraries).
- Information that could be gathered without a warrant or in breach of a data privacy statute.
- Available through the Surface Web (*e.g*., social networking sites and all search engines may access), the Deep Web (including historical database information), or the Darknet.
- Information that is discoverable by specialized search engine operators, such as Google dorks or Bing search operators.
- Data available in public meetings/discussions or that is audible to the average listener.

## What is iso 27001?

ISO/IEC 27001: 2013 is an international specification that defines standards for handling information security within an organization. It allows organizations of all types to maintain the security of financial records, intellectual property, employee details, and information delegated to third parties.

This section will look at how ISO 27001 (or the ISO/IEC 27001:2013 standard) can be used to develop, implement, maintain, and continually expand an Information Security Management System (ISMS). An ISMS is a comprehensive method to handling an organization’s crown jewels (*e.g*., precious properties and data) and confidential documents to ensure continued protection through risk management strategies. Additionally, there are three primary security objectives within an organization’s ISMS:

- Privacy—accessibility to details is restricted to approved employees.
- Authenticity—only approved staff can change the data.
- Accessibility—the records must be readily available to designated employees at all times.

### Security controls (Annex A)

ISO 27001’s Annex A, or the controls portion, includes a list of 114 industry-standard protection controls or protections divided into 14 divisions and classified according to the categories listed as shown in Table 2:

**Table 2:** Security controls.

Data security policies: Establishing great information and rules for cybersecurity in compliance with company needs and relevant laws and regulations.
Data protection organization: Creating a framework for facilitating and monitoring the application of cybersecurity.
Performance appraisal: Checking that staff and vendors are mindful of and fulfill their cybersecurity obligations before, during, and after work and recognizing their obligations and are fit for the positions for which they are being chosen.
Integral gain: describe and specify the necessary protections, for example, the avoidance, alteration, elimination, or loss of information contained in newspapers and any unwanted information.
Control monitoring: Ensure that access to information collection and processing facilities is limited and that permitted access to systems and resources is guaranteed and prevented from unauthorized access.
Cryptography: checking that cryptographic knowledge is correctly and efficiently used to preserve information security, reliability, and honesty.
Environmental and physical safety: Avoiding unwanted entry to the organization's material and informational computing infrastructure, disruption, and conflict.
Protection of services: Providing proper and safe information management services activities.
Surveillance of information exchange: ensuring network safety and related information management services and ensuring security for information sent within a company and to every external body.
Technologies procurement, growth, and servicing: Establishing that computer management is integrated with the lifecycle of information systems. This provision often applies to computer structures that deliver utilities over open networks.
Performance framework: Making sure the company resources made accessible to suppliers are secured.
Control of computer protection incidents: Providing a transparent and efficient approach to risk management, including correspondence with threat intelligence and vulnerabilities.
Components of operational processes relating to digital technology: Integrating cybersecurity continuity into an organization's Business Continuity Management (BCM) processes.
Enforcement: Prohibiting violations of civil, constitutional, administrative, or statutory responsibilities and protection provisions relating to information security, including compliance with applicable and compliance issues and conducting cybersecurity assessments.

### Becoming ISO/IEC 27001:2013 Compliant: Who, When, Where, Why, How

- **Who**: ISO/IEC 27001:2013 ideal for an organization that needs to enhance its knowledge technology by implementing industry-wide best-recognized standards for surveillance?
- **When:** ISO/IEC 27001:2013 will be introduced and approved at any point. The company can opt to enforce the norm and credential later when regulations need it, raising consumers’ and clients’ confidence in a move at the same time.
- **Where:** Both organizations can use the definition regardless of whether they are for-for benefit, nonprofit, or public-based entities.
- **Why: ISO/IEC 27001:2013 will** improve protection by applying it essentially. It lets companies comply with regulations, attracts clients by reassuring them about the protection, lowers expenses, and organizes an efficient framework for data security processes and regulations. See Table 3.

**Table 3:** ISO/IEC 27001:2013 compliant.

How: An organization that wishes to enhance its security management framework by adhering to ISO/IEC 27001:2013 will perform the following activities:
Gap review: As the first move toward implementation, either internally or with the assistance of an impartial information technology specialist, a gap analysis is conducted. A distance appraisal enables the company to thoroughly comprehend which standards and controls it complies with and which it does not.
Regeneration: If a company is not per specific standards or controls, it will improve the personnel, procedures, and technology to become compliant.
Definition, Track, and Audit: The success of the ISMS must be continuously analyzed and checked for efficacy and enforcement and to find opportunities to enhance internal procedures and controls.
Assessment process: A practical understanding of the lead audit procedure is needed for the ISMS at scheduled intervals and is also critical for champions responsible for adopting and enforcing ISO/IEC 27001:2013 enforcement before performing a certification audit by an external auditor or agency approved to approve and classify an entity as ISO/IEC 27001:2013 compliant.
Certified and identity verification: During the Stage One certification audit, the inspector will determine if the report complies with the ISO/IEC 27001:2013 specification and will highlight any areas of non - conformity and future management system change. After incorporating the appropriate adjustments, the company would be eligible for a Stage Two registration check. The auditor would perform a comprehensive evaluation of the organization's conformity with the ISO/IEC 27001:2013 standard during a Stage Two audit.

### ISO 27001’s advantages

ISO 27001 is one of the most frequently accepted standards for data processing. Globally recognized is certification of the Benchmark by an independent accredited entity. In the last decade, the number of credentials has increased by more than 450%.

By adding the Standard, one can maintain consistency with information security management systems enforced by legislation such as the EU GDPR (General Data Protection Regulation) and the NIS (Network and Information Systems) Regulations. This reduces the level of risks relating to data breaches.

- Ensure the security of your files, regardless of their location
- Risk savings associated with computer management
- Enhance the corporate atmosphere
- Improve the threat resistance
- Anticipate and react to emerging cyber threats
- Comply with statutory requirements.

### ISO 27701 privacy regulations

Privacy security in a rising wired environment is a necessary expense. New privacy laws, such as the General Data Protection Regulation (GDPR), enforced by authorities, force businesses to act. ISO specifications such as ISO/IEC 27701 can help the company fulfill specifications and handle privacy threats relating to sensitive details (PII). ISO 27701 Privacy Information Management System (PIMS), expansion to ISO 27001 Information Security Management System (ISMS), will help the organization fulfill compliance standards and handle privacy threats associated with Personally Identifiable Information (PII). Implementing a management framework compatible with ISO/IEC 27701 and ISO/IEC 27001 would allow the business to satisfy GDPR’s privacy and information security standards and other data protection regulations. GDPR allows organizations to take adequate technological and operational steps (including protocols, procedures, and processes) to safeguard their records. Microsoft’s involved committee member.

Julie Brill, Corporate Vice President and Deputy General Counsel for Privacy and Regulatory Affairs at Microsoft, said:

“We thank the ISO/IEC Technical Committee for introducing this innovative privacy standard so that companies of all sizes, territories, and sectors can easily protect and monitor their data. As the next chapter in Microsoft’s dedication to extending protections under the General Data Protection Regulation of the European Union to our customers internationally, Microsoft Azure and Office 365 will enforce the PIMS standard and support our customers and collaborators in implementing this interoperable model.”

#### ISO 27701 CERTIFICATION BENEFITS:

- **Assist in ensuring conformity with privacy legislation—**such as the European Union’s General Data Protection Regulation (EU GDPR) and domestic privacy laws and regulations such as India’s Personal Data Protection Act (PDPA).
- **Instill trust in partners and consumers—that you adhere to the strictest protection requirements when handling privacy threats associated with PII.**
- **Well-defined positions and duties—**for PII controllers and PII processors that are accountable for PII processing.
- **Minimize costs –** with sensitive systems being disrupted and financial damages associated with a violation.

## Problem statement

The automated forensic methodologies do not provide functionality for utilizing OSINT knowledge since most models are built on obtaining physical hardware only. Adults and teens both are embracing new technology and the usage of the Internet. One of the fastest-growing online collaboration methods is sharing personal details to make sure the researchers are aware of all this knowledge to be made accessible to them; a system needs to be built for OSINT techniques such as social media.

### Objectives and research question

This analysis discussed the following research question: Will OSINT be used for automated forensic investigation, and is the information gathered permissible as proof in a legal proceeding? To resolve this problem sufficiently, the following sub-objectives should be approached:

**Q.1.** Determine what evidence obtained from social media and Open-source intelligence (OSINT) can advise a forensic analysis officer in an investigation.
**Q.2.** Develop a system that digital forensic experts may utilize when performing an inquiry that involves using social networking services in part or entirely to collect evidence.
**Q.3.** Develop standards on what comprises a social networking platform and the kinds of knowledge or facts accessed from these pages.
**Q.4**. Discuss any shortcomings or difficulties discovered by a digital forensic investigator when performing a completely or partially forensic inquiry involving social media platforms.

In answer to the research query, the researcher hypothesizes that the knowledge collected from OSINT has valid significance and may be used to support cyber forensic experts.

## Research methodology

This section explains the study’s strategy and methods. The motives for using a conceptual framework are discussed, as is the methodology for obtaining a random sample. Additionally, this paper describes the process and resources used to capture and interpret the results.

### Investigative process

**Figure 5:**
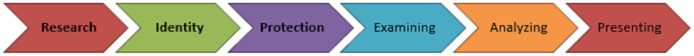
Research investigation process.

In 2001, as part of the DFRWS, a forensic protocol was established that contained the following types of steps that experts believe should be taken while conducting a remote forensic analysis (Palmer, 2001): Casey (2011) updated the most often used steps for performing a thorough automated forensic examination and recommended that the following steps be taken see Fig. 5.

### Research strategy

It attempts to illustrate online analysis and gather information research on OSINT and social networking techniques as data. Rather than treating humans as static units, we consider their test subjects that merely respond to stimuli; we have been experimenting on them. Other responses were offered in the report and, for instance, on the subjects of the findings. However, it is all research-based, so there is no way to quantify most of the data. The survey findings were examined, and a first quantitative analysis was undertaken, followed by a qualitative evaluation of the outcomes (Saunders, Lewis & Thornhill, 2009). when phenomena can be viewed as just as themselves, cross-over phenomena can be used for studies or studied over a single timespan (Saunders, Lewis & Thornhill, 2009, p. 155).

### Data collection and analysis

An electronic sample of digital forensic professionals delivered the primary data used in the questions used in this research. Participants may participate electronically; much of the survey is completed by email or the Internet. The central part of the survey was developed using rating questions, which are commonly used to solicit views. Using a Likert-style scale, the researcher was able to conduct detailed observations and interviews, the results of which were qualitative. When expanding the questionnaire, respondents were asked to provide an answer or explain each assertion. They were then asked to answer the agreement or disagreement with statements or statements, respectively (Saunders, Lewis & Thornhill, 2009). More questions were asked to obtain additional details when this response was provided to clarify previous answers.

The survey was split into three sections: longitudinal data collection, social network monitoring, and OSINT collection. The survey’s first segment focused on longitudinal results. The details obtained from the questionnaires will provide the researcher with information about the participant’s qualifications, the number of years of experience performing digital forensic investigations, the field they currently operate in, and, eventually, their position in digital forensics. The second segment included concerns about social media use. Participants were asked questions about utilizing social media and how they use it while performing automated forensic investigations. This section is essential for the researcher. The comments can help them gauge the researchers’ understanding of how social networking is used and how users feel during digital investigations. Inquiries inside the third section included OSINT for the third time for the study; survey respondents were asked whether they used OSINT in addition to or whether it was done as part of an automatic investigation. Analysis techniques were derived from the information which was required for OSINT use.

### Survey participants

Only digital forensic professionals were included in the study’s sample category. A sample questionnaire was emailed to 35 respondents. This sample population specifically consisted of those who have attended advanced forensics seminars. All of them have already shown their readiness to engage in the survey research. Despite the researcher’s awareness of all respondents, the researcher performed the 20 questionnaires anonymously. Just 9 completed electronic questionnaires were sent out of the original 75 sent. The test sample was entirely voluntary whenever someone got in touch with them *via* text, they were informed of the following:

- The sample questionnaire will be confidential, and the identity of the applicant will not be revealed in the study;
- They might avoid the research altogether if the questionnaire were too distressing; and
- All participants would obtain the results upon request.

### Participants’ demographic information

Tables 4 and 5 show the demographic questions and characteristics of the study population. We had information regarding their educational institution, working area, and the number of years they have been in the field for respondents. They said that they are in charge of a digital forensic project with different degrees and career titles.

**Table 4:** Demographic questions.

1. What is the highest level of Education?
2. How many years of forensic experience do you have?
3. In which industry are you currently working?
4. Which role best describes you?
5. Country of your performance?
6. Are you comfortable with social media investigations?
7. Which social media app do you use most frequently?
8. Define OSNIT in one line?

**Table 5:** Respondents’ demographic information (*n* = 9).

Participant	Location	Highest tertiary qualification achieved	Industry	Current digital forensics role	Are you comfortable with social media investigations?	Years of digital forensic experience
1.	Australia	Honor degree	Education	Consulting	Yes	3–5 years
2.	Kenya	Bachelor’s degree	Finance	Corporate Investigator	Very much	8–10 years
3.	South Africa	Master’s degree	University	Forensic expert	Extremely high	10–12 years
4.	New York	Engineering degree	Software agency	Consultant	Neutral	4–5 years
5.	Britain	Diploma in computers	Government agency	Corporate investigator	Very comfortable	6–8 years
6.	India	Professional certification	Consultant	Insurance	Neutral	3–5 years
7.	Singapore	Diploma	College	Computer expert	High	6–8 years
8.	USA	Bachelor’s degree	Information Technology	Forensic expert	Pretty sure	4–5 years
9.	Germany	Doctorate’s degree	Law Enforcement	Cybercrime expert	Extremely high	9–12 years

In our survey, a various questionnaire was asked to the participants, and some the questions are given below:

### Analysis of survey questions

Usage of Social Media to obtain Specific Information On a scale of 5 (strongly agree) to 1, participants were asked to evaluate the extent of their belief that social media sites could help modern forensic investigators recognize certain types of information (strongly disagree).

**Figure 6:**
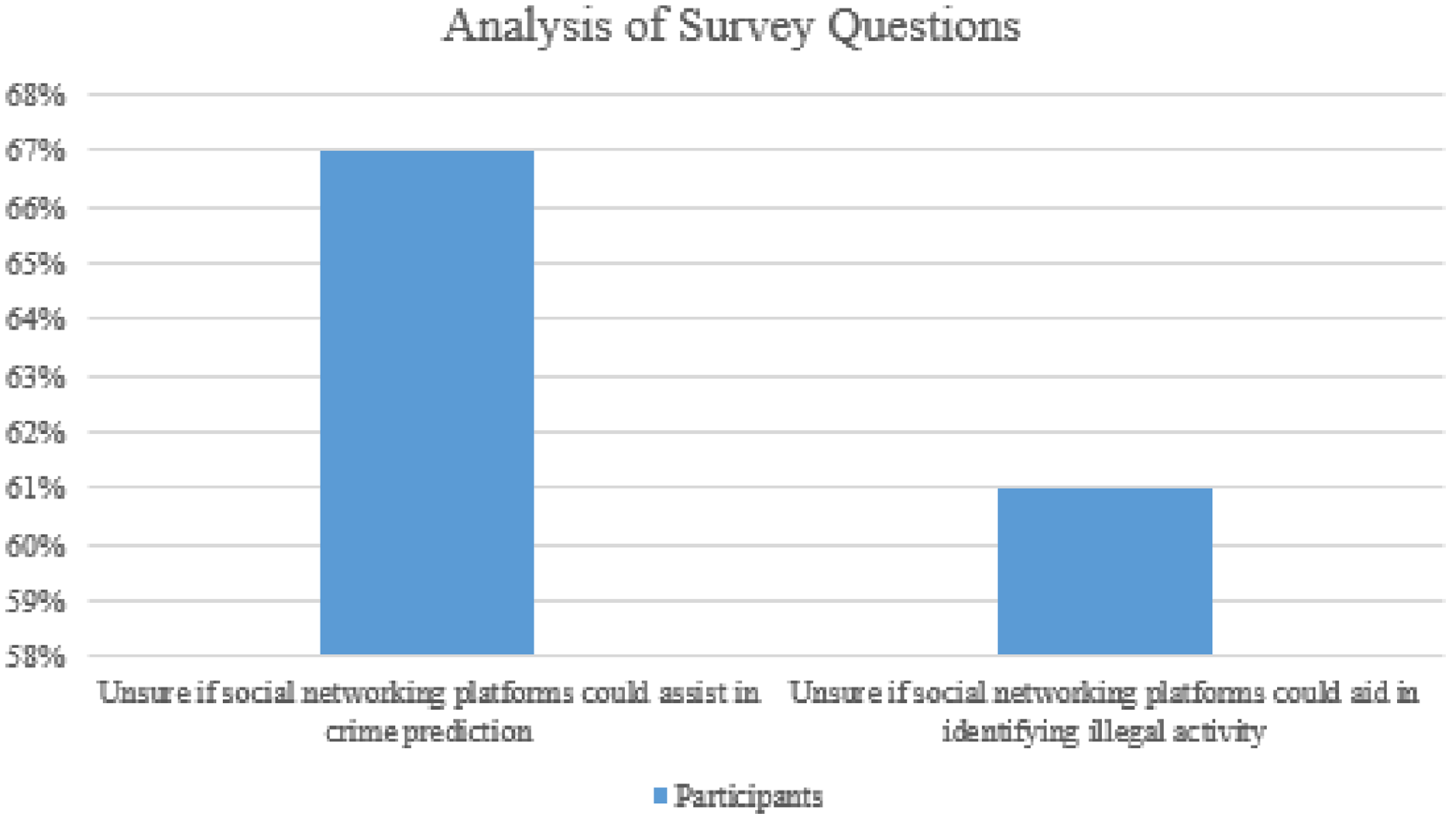
Analysis of survey questions.

Responses (*n* = 9) to whether social networking platforms can help digital forensic investigators obtain specific types of evidence. Participants generally agreed that social networking sites could assist digital intelligence officers in developing all but two kinds of evidence presented (Fig. 6).

- 67% of respondents were unsure if social networking platforms could assist in crime prediction.
- 61% of participants were unsure if social networking platforms could aid in identifying illegal activity.

According to the LexisNexis (2012) Study, the top three applications of social media for criminal investigations are “identification of persons and places, the discovery of criminal behavior, and finally, proof collection” (LexisNexis, 2012).

According to a law enforcement official surveyed by LexisNexis in 2012, social networking sites aided crime prevention. Through launching an inquiry through Facebook, the officer believed they had averted a “Columbine-style shooting.” There was sufficient evidence that the warnings were genuine, and further investigations revealed that a student was in the process of buying weapons and intended to harm others (LexisNexis, 2012).

### My social networking sites’ tools

The availability requirements attempted to decide which digital forensics specialists used while conducting social media digital forensic analyses.

Responses (*n* = 9) to which techniques are utilized when conducting automated forensic investigations *via* social media. Internet Evidence Finder (IEF) is a proprietary piece of software and, using the information available over the Internet, is effective in restoring thousands of Internet-related objects. And is used by 61% of participants in a poll. IEF is a dependable tool since it can extract information from social networking sites, webmail, online computing, web traffic, and many other sources.

On the Internet, there is a tool for locating evidence. Vulnerabilities in Google The Malthusian theory Network examinations Python scripts for web crawlers Application for searching details using Cacheback Bulk Extractor Google hacking, for example, entails utilizing the Google search engine in conjunction with various search operators that have definitions exclusive to the Google search engine (Long, 2008). When a search operator such as “filetype: pdf” is used, Google can return results that include websites that use the text extension pdf. When doing an Internet search for a particular pdf text, this search is beneficial.

**Figure 7:**
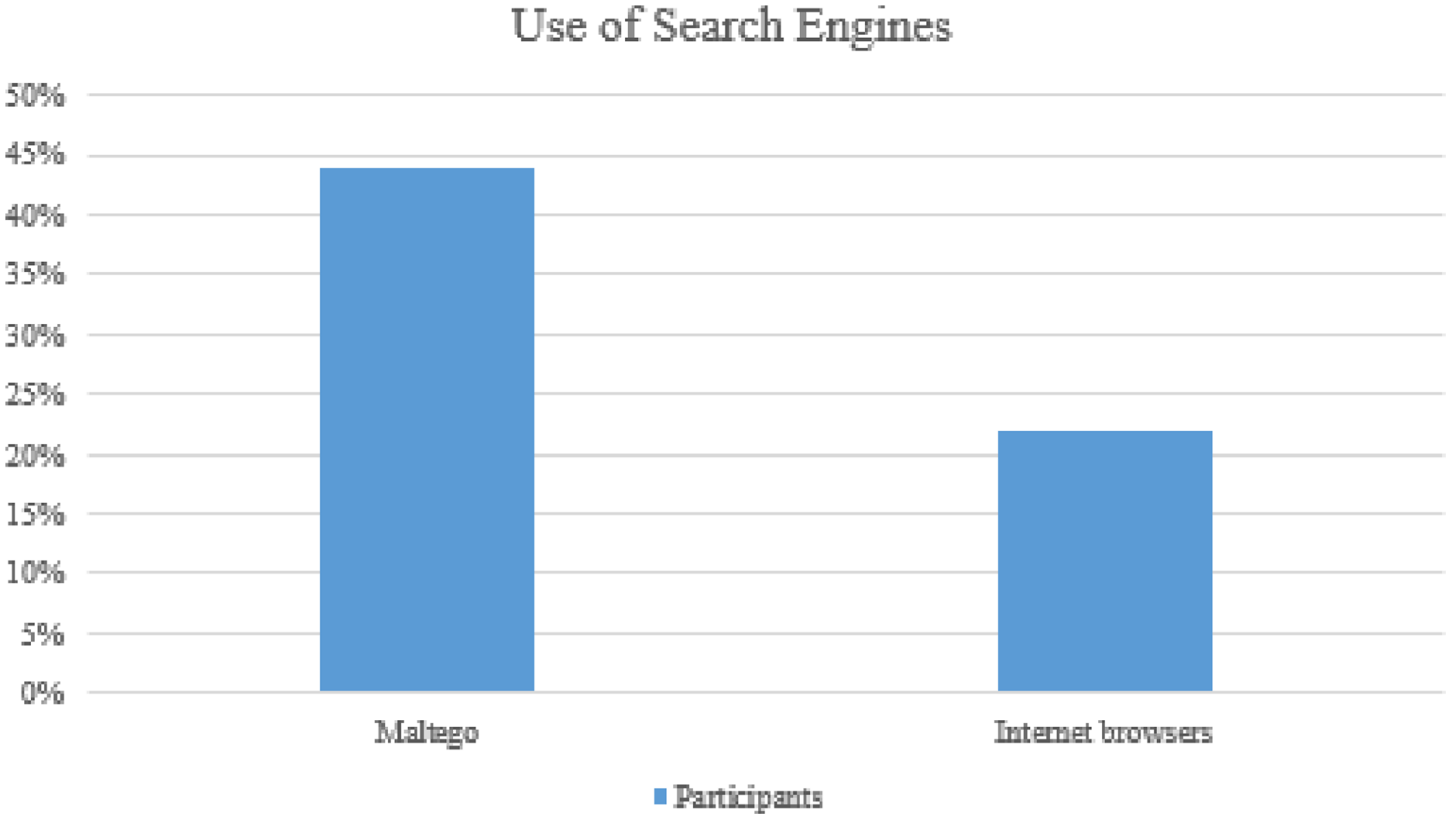
Use of search engines.

According to Bradbury (2011), search engines are a valuable instrument, and Google hacking is a valuable technique. Another useful Google trick is “cache: website,” which returns a cached version of a website, applicable when searching for an outdated version of a website. Half of those interviewed said they had used Google Hacks. Maltego, an OSINT and forensics app, is used by 44% of respondents (Fig. 7). Maltego is a program that analyzes and visualizes data relationships. Running data from the social networking tool LinkedIn from Maltego, for example, produces results such as memberships in other social networking sites and contact details, depending on which the LinkedIn profile user has supplied this information (Bradbury, 2011). Maltego’s capacity to browse the Internet for important details such as email addresses is another useful function (O’Connor et al., 2010). When a user enters a name and surname into the Maltego email search feature, the site is searched for all email addresses that include the entered name and surname.

Netanalys, a data collection and analysis platform for different Internet browsers, 22% of those surveyed. The program is valuable since it can download Internet history and cache data from a variety of browsers. Python is known as a hacker’s programming language because of its ease of use. TIC serves as an excellent software for the implementation and dissemination of specific tactics. It is often considered compliant with various specialized platforms, allowing every army mod to build its levels or mods (O’Connor, Stewart & Smith, 2013). According to respondents, one-third of those who use Python for automated tools in-network investigation use social networking as their primary method. As done in a script that uses the Twitter APIs for data recovery in digital forensics, retrieve helpful knowledge is used for testing and scraping Twitter as the details contained in Twitter data extraction buttons (API).

Python scripts may be used to retrieve any content, including users’ tweets and retweets and geographic position (O’Connor, Stewart & Smith, 2013). In Appendix, there is a Python script for the python method that searches Twitter for the positions of new and retweets. Crawdads may also be referred to as web spiders or scrapers, based on the feature under consideration. Clough defines search engines as browser software that reviews different websites, retrieves details, downloads files, positions them in a database section, and stores all information on internet pages.

Web crawlers, like web spiders, are often used by hackers to obtain email addresses from the Internet. Only 1 in 7% of respondents found evidence of web-seeking software to be relevant during a digital forensic investigation. Cashback is an internet investigation program for multilingual web searching, analysis, originally called Expando, is now known as Cacheback, and can even look for security details in web browsers. XIT can retrieve the background of several web browsers and caches it to be presented on a new page and helps the investigator display the Internet history connected to it. Approximately two-in-of-ten respondents answered, “expand upon,” or two out of every 10 responded, “add to” An email address and username.

URL gathering utility is a function that can also extract account information for automated forensic purposes. The bulk extraction tool may also accept data from a hard disk (an investigator’s hard drive, a memory card, or camera) and media connected to it. Bulk extractor’s most significant power resides in its ability to inspect numerous drives or images, which is why the application uses multiple scanners (Garfinkel, 2013). only 11% of the respondents said they used a bulk extractor when involved in automated forensic examination.

**Figure 8:**
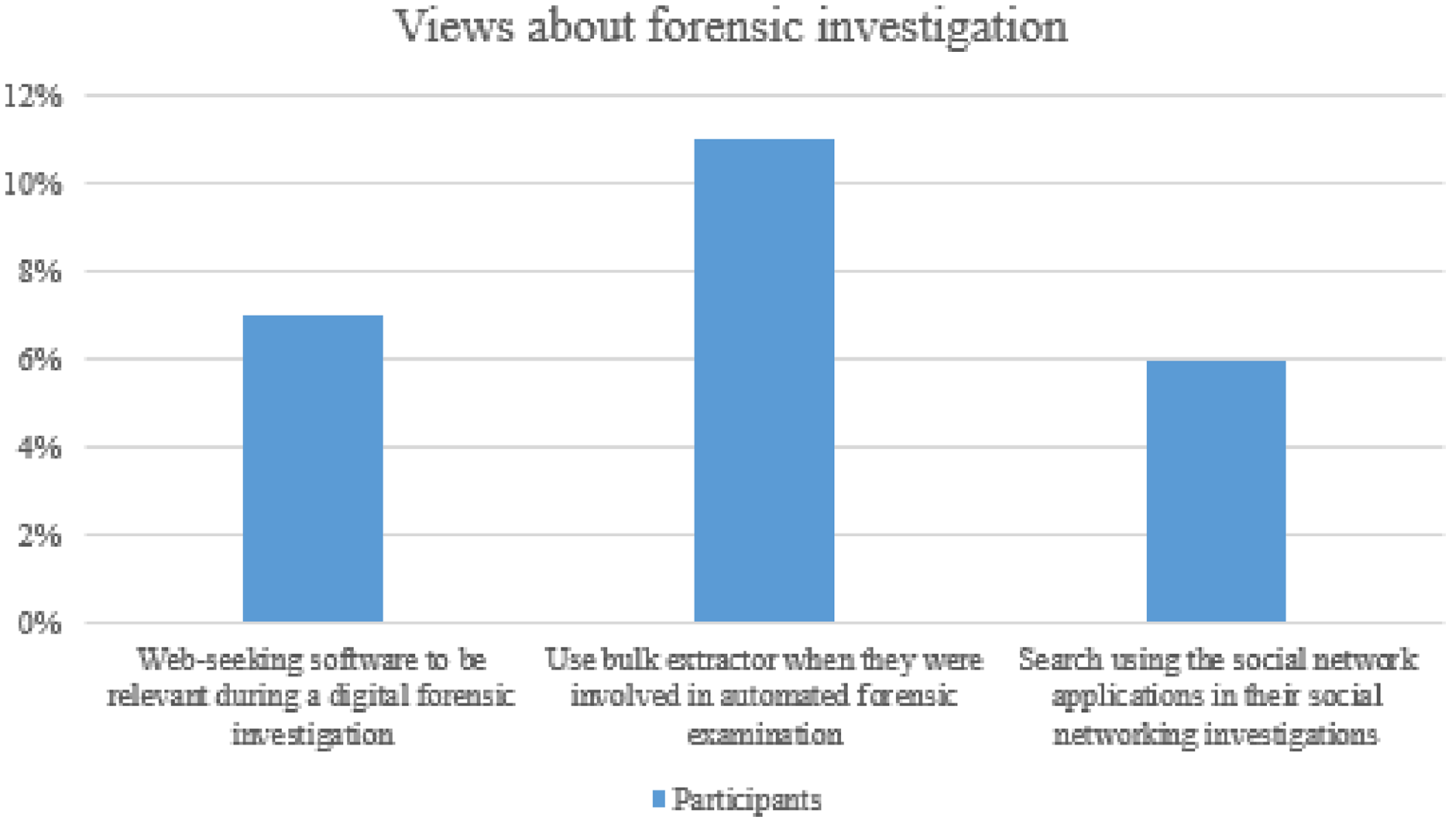
Views about the forensic investigation.

Although there are several methods of using Twitter’s open-source elements, you can make an application around a system that meets the specifications of the Twitter searchable terms of use so long as the original developer stays in compliance with the terms of use. Malte is proficient in API (or application programming interface) requests. The functionality in Malgo helps anyone find tweets on the Internet by providing Malte consumers with the user’s current location, tweet data, and retweet information. Search results for an individual or the Twitter photos, media often apply to their Twitter account are made available for Malgo’s followers through the platform’s image search feature. The API that allows users to retrieve tweets and locations also creates scripts that retrieve tweets and positions. Only 6% of respondents mentioned they have ever searched social network applications in their social networking investigations (Fig. 8).

### The most famous social networking sites

**Figure 9:**
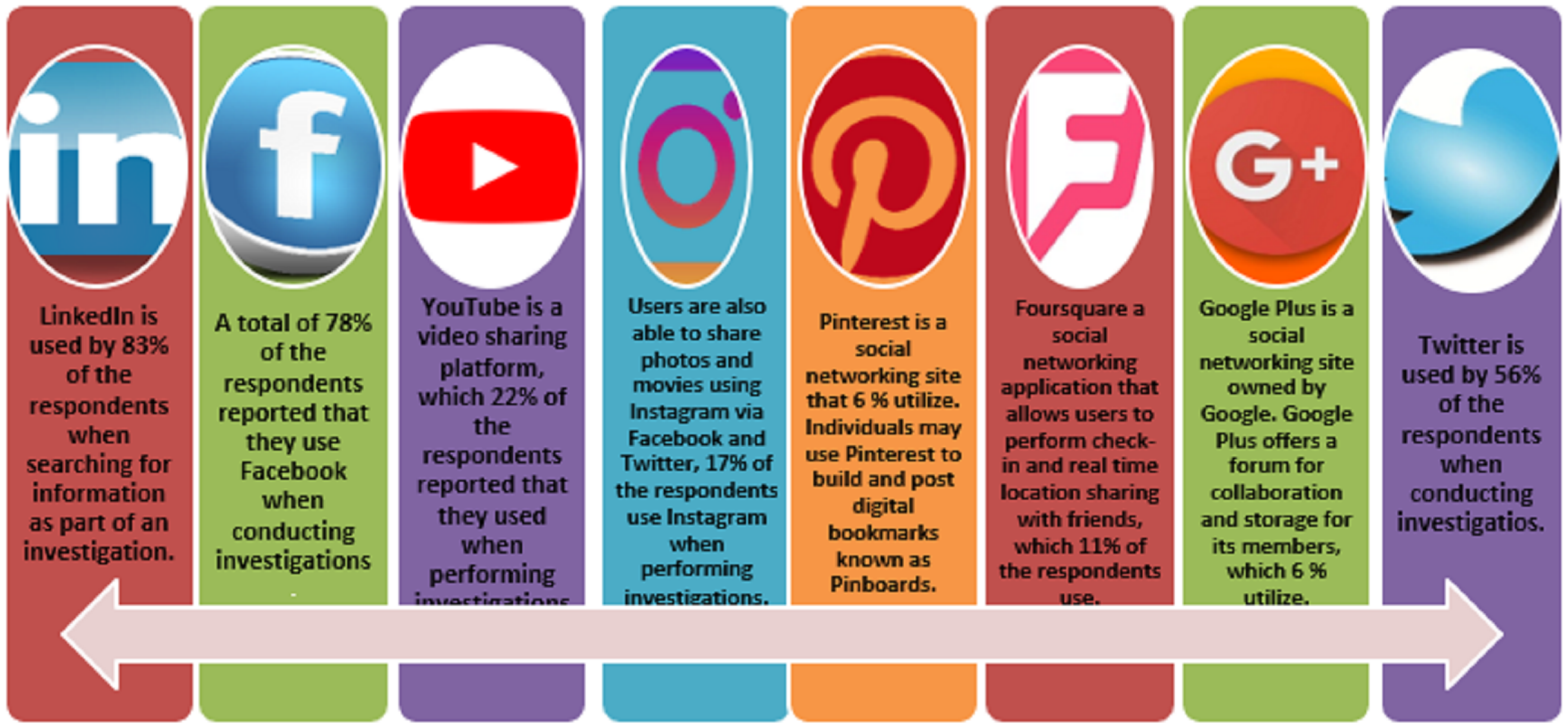
Internet sites and social networking.

How do prosecutors use social media to increase their understanding of the case’s background to find and obtain and organize help material for their investigative details? Respondents (*n* = 9) were asked which social network networks they most commonly utilized while conducting an inquiry (Fig. 9).

A total of 83% of respondents use LinkedIn to collect evidence for an inquiry. Aside from the obvious things, including an email address, geolocation, and marital status, a LinkedIn profile provides a wealth of details regarding the person. LinkedIn is primarily a professional networking platform; it includes details about an individual’s job title, previous employer, and educational institution. Additionally, it suggests potential associates (Bradbury, 2011).

As of July 27, 2014, there were roughly 1.32 billion Facebook members, with an average of 4.75 billion things posted every day, according to Facebook’s main facts12. A total of 78% of those polled claim they use Facebook for study purposes. When performing interviews for the (LexisNexis, 2012) report, law enforcement officers found that the most often used personalized social networking platforms, such as Facebook and YouTube, are personal social networking accounts. LexisNexis (2012), 56% of respondents used Twitter in their inquiries. Twitter can show who follows a particular user associated with a Twitter handle and follows the Twitter account itself. This information is especially beneficial if an individual’s interactions with other individuals or organizations are being monitored. Twitter was used in 2011 to rally a group of Twitter users to plead for assistance in sweeping up the streets after the 2011 London Riots. YouTube is a popular website for video sharing where 22% of users registered. A participant in the LexisNexis (2012) police study confirmed that YouTube helped in the active arrests of gang members have produced a recruitment video and increased gang-related activity Thesaurus.

Instagram is a social networking site that a web application on smartphones can access. Users may use Instagram to post videos and images. Users of Instagram would also share photographs and videos on Facebook and Twitter; 17% of respondents use Instagram for testing purposes. Eleven percent of respondents use Foursquare, a social networking platform that enables users to log in and share their current position with others. Google Plus is a social networking site run by Google. Google Plus, which 6% of respondents use, offers a network for networking and storage. Pinterest is a social networking site that 6% of respondents utilize. Individuals can build and post Pinboards, which are digital bookmarks, on Pinterest. A Pinterest account can be connected to a current Twitter or Facebook account, allowing users to update their Facebook and Twitter mates if they add a new pin to their Pinboard.

## Discussion of results

The study aimed to see whether social media and OSINT were effective in assisting digital investigators. According to a survey, most respondents believe that social networks and open-source intelligence will aid modern investigations in this endeavour. Second, although the plurality of respondents (89%) agreed that knowledge gathered *via* social media is useful, only slightly more than half (56%) agreed that information acquired *via* OSINT networks is valuable. The two findings can be explained by respondents’ lack of a clear concept of OSINT. Social networking is a distinct type of OSINT or because responders are more comfortable in digital forensics, including their minds.

Additionally, suppose participants do not often use social networking services. In that case, they can be confused about their different uses and features. These include guidance for using social media sites and identifying which kinds of knowledge can be gathered from these sources.

This aim is achieved by verifying their most widely used social networking accounts during inquiries. LinkedIn is 44% the most often used social networking platform. One possible reason for this decision is that browsing LinkedIn’s publicly available user profiles may not include a legitimate LinkedIn user. Though LinkedIn user accounts are freely accessible, the information shown is dictated by the privacy settings chosen by the proprietor. Because LinkedIn is marketed as technical networking and social networking tool, profile owners may leave their profiles exposed while searching for new job opportunities. A LinkedIn profile contains the following types of information: profile picture, work history, group participation interests, education level, and any personal websites added by profile consumer. Several respondents mentioned utilizing social networking sites to supplement their selection efforts. The research results also suggest that only a limited proportion of respondents felt comfortable initiating social network inquiries. This lower take-up rate could result from a lack of formal planning and guidelines for performing such investigations.

There may also be concerns about social networking knowledge being treated as secondary rather than proof, as digital forensics is primarily focused on presenting indisputable details about the data being analyzed, primarily when the data is intended for use in court. The proof will be considered facts if, through a warrant, the material is retrieved from the social network, and the documentation is collected forensically, as the respondents suggested. The respondents (89%) felt creating and making an OSINT toolkit available would be helpful. Besides, 83% of participants accepted that designing an OSINT system for inquiries would be helpful. Because of the extensive usage of social networking sites, respondents suggest an OSINT toolkit and system will be helpful for investigations. Also, respondents are possibly conscious of the abundance of knowledge accessible across numerous OSINT networks but are looking for advice or a tool to help them conduct OSINT investigations. Investigators could have preferred to use established tools and techniques, resulting in a need for a system or administrative scheme.

The internal or external digital investigation that is commissioned to be carried out requires legal permission. In reality, each level of digital research should be authorized, and therefore each procedure must be authorized. Authorization for law enforcement investigations sometimes necessitates a search warrant or other legal permission based on adequate evidence or suspicion. In most cases, search warrants are unnecessary for business occurrences if adequate privacy rules are in place (Montasari et al., 2019). When a suspect is interrogated to provide a password to a system under investigation, a digital investigation is dependent on a physical investigation (Valjarevic & Venter, 2015). There is a need to include interaction with the physical investigation because defining the relationship between a digital investigation and a physical investigation is required to preserve the chain of custody, preserve the integrity of the digital evidence, protect the digital evidence from damage, and ensure an efficient investigation.

## Future research and recommendations

There is a lot of analysis to be done in automated criminal operations using OSINT and, more importantly, social networks. In this instance, Casey’s proof scale could include OSINT. An OSINT source may be used as a reference point for gathering preliminary research.

The drawback of this survey is the insufficient number of remote forensics experts who participated. Despite sending two follow-up emails demanding the recipients’ inclusion in the study, only nine replies were obtained from a total of 35 digital forensic practitioners. Despite this limitation, the paper implies that the research reported in this paper is nevertheless relevant in the long run, as the information gained from forensic practitioners was merely used to describe the *status quo* of OSINT use in digital forensic investigations as a starting point for defining the framework for gathering evidence. Despite this limitation, the author argues that the research reported in this paper is nevertheless relevant in the long run, as the information gained from forensic practitioners was merely used to describe the *status quo* of OSINT use in digital forensic investigations as a preliminary step for defining the framework for finding data.

## Limitations

The major constraint to gathering open-source knowledge is the overwhelming amount of digital data, the ever-growing number of information outlets, and the data’s unreliability. As the advanced transition continues to integrate all facets of work and life, delving through this massive mass of ordered and unstructured public records becomes prohibitively tricky and repetitive. As a result of tremendous technological advancements and the breadth of Internet-based exchanges, OSINT has developed into a critical component of any organization that wishes to examine the reach of online data that could either cause harm to the enterprise or allow its successful synergy with exceptional expertise.

## Conclusion

From the study findings in this paper, it has been estimated that social media and OSINT (open-source intelligence) outlets provide a rich supply of knowledge that helps investigators do their work. The paper’s second goal was to develop a system that could be utilized to perform inquiries concerning knowledge obtained from social networking platforms. As mentioned in this document, three of the paper’s goals have been fulfilled. Because of the increased usage of the Internet, innovative advanced forensic methods have been essential. Much research on digital forensics and conventional forensics is analyzed in the abstract. Are unsuitable for OSINT proof (Richard & Roussev, 2006). because of the quantity of publicly accessible personal information in social networking, the study results showed that a system for using OSINT is valuable. No guidance or legislation exists for organizations to conduct automated investigative examinations into social networking apps (Taylor et al., 2014). The study showed that valuable knowledge is present on social networking platforms, and a guide was recommended for digital forensic investigators. Based on the OSINT details, a digital forensic investigation system was created, all the framework’s principles were established. Each theory was explained in detail. Finally, a series was added to each theory outlining behaviour and available means. This guide presented OSINT investigators with a blueprint for performing their forensic studies. Additionally, the researcher also presented guidelines for each framework phase. The main goal of the new system was to ensure an evidence-based procedure that OSINT researchers could adopt in the future.

## Supplemental Information

10.7717/peerj-cs.810/supp-1 Supplemental Information 1 Questionnaire.

10.7717/peerj-cs.810/supp-2 Supplemental Information 2 Answers to the questionnaire.

## Additional Information and Declarations

### Data Availability

The following information was supplied regarding data availability: The raw data are available in the Supplemental File.
