# Supplementary material for: The effect of ISO/IEC 27001 standard over open-source intelligence
Source: PeerJ Comput Sci. 2022 Jan 6;8:e810. doi: 10.7717/peerj-cs.810 (PMC8771761; doi:10.7717/peerj-cs.810)
Supplement: Supplemental Information 1 [file peerj-cs-08-810-s001.pdf]

Survey's questions

Q1) What is the highest level of Education?

Q2) How many years of forensic experience do you have?

Q3) In which industry are you currently working?

Q4) Which role best describes you?

Q5) Country of your performance?

Q6) Are you comfortable with social media investigations?

Q7) Sort the social media app do you use most frequently.

Q8) Define OSNIT in one line?

Q9) Do you believe that social media sites could help modern forensic investigators recognize certain types of information?

Q10) Can social networking platforms assist in crime prediction?

Q11) Can social networking platforms aid in identifying illegal activity?

Q12) which techniques are utilized when conducting automated forensic investigations via social media.

Q13) Do you think creating and making an OSINT toolkit available would be helpful?

Q14) Do you think designing an OSINT system for use in inquiries would be helpful?
